# Emotional judgments of scenes are influenced by unintentional averaging

**DOI:** 10.1186/s41235-020-00228-3

**Published:** 2020-06-11

**Authors:** Yavin Alwis, Jason M. Haberman

**Affiliations:** The Department of Psychology, Rhodes College, 2000 N Parkway, Memphis, TN USA

**Keywords:** Ensemble perception, Scenes, Valence

## Abstract

**Background:**

The visual system uses ensemble perception to summarize visual input across a variety of domains. This heuristic operates at multiple levels of vision, compressing information as basic as oriented lines or as complex as emotional faces. Given its pervasiveness, the ensemble unsurprisingly can influence how an individual item is perceived, and vice versa.

**Methods:**

In the current experiments, we tested whether the perceived emotional valence of a single scene could be influenced by surrounding, simultaneously presented scenes. Observers first rated the emotional valence of a series of individual scenes. They then saw ensembles of the original images, presented in sets of four, and were cued to rate, for a second time, one of four.

**Results:**

Results confirmed that the perceived emotional valence of the cued image was pulled toward the mean emotion of the surrounding ensemble on the majority of trials, even though the ensemble was task-irrelevant. Control experiments and analyses confirmed that the pull was driven by high-level, ensemble information.

**Conclusion:**

We conclude that high-level ensemble information can influence how we perceive individual items in a crowd, even when working memory demands are low and the ensemble information is not directly task-relevant.

## Significance

If we encounter someone who appears upset, the appropriate response might be to express sympathy or offer support to that individual. Successful interaction with an individual depends on a veridical interpretation of their emotional state. However, can the surrounding scene context influence an observer’s judgment about an individual’s emotional state? If, in the above example, we saw that sad individual in the context of a party where everyone appeared to be happy, would that influence how upset we perceived that person to be? The current experiments suggest, yes, the interpretation of an individual’s emotional state is biased toward the emotional state of other nearby people, even when that information is irrelevant. This suggests that ensemble information, while a generally useful algorithm for compressing redundant information, can have unintended, and perhaps undesirable, consequences for the perception of one’s emotional state.

## Background

Conscious visual perception tends to be a singular and unified experience, despite an overwhelming amount of incoming visual information. Although how perceptual unification is achieved remains unresolved, psychologists have identified a number of heuristics that may provide some insight into this challenge (Helson, 1933; Wertheimer, 1923). For example, the Gestaltists conceived of a number of simple rules for determining the likelihood that something would be perceived as a singular surface (e.g., good continuation, proximity, good form). These rules may be used to guide the successful segregation of textures and surfaces (ala Marr, 1982), a likely prerequisite to a unified perceptual experience. Convergent evidence comes from neurophysiology, which has revealed a number of convergent cellular mechanisms that map onto the Gestalt rules, such as border ownership cells (Zhou, Friedman, & Von Der Heydt, 2000), cells that respond to subjective contours (Von der Heydt, Peterhans, & Baumgartner, 1984), and cells that respond to complex shapes and contours (Tanaka, 1996), forging an important link between psychophysical and neuroscientific approaches.

These mechanisms are still insufficient, however, to support a wholly *veridical* perceptual experience. The shared ‘grand illusion’ of visual completeness (Noe, Pessoa, & Thompson, 2000) belies how impoverished our visual consciousness truly is (Luck & Vogel, 1997; Rensink, ORegan, & Clark, 1997; Simons & Chabris, 1999). We must appeal to additional heuristics to account for a unified perceptual experience in the face of limited representational fidelity. The heuristic most germane to the current study is the phenomenon of ensemble perception, the visual system’s tendency to extract an average representation from redundant information within a scene (Ariely, 2001). The ensemble system summarizes a complex scene efficiently and succinctly, but at the expense of detailed representations at the individual item level (i.e., it creates the experience of completeness despite limited information). Ensemble perception is ubiquitous, operating at multiple levels across the visual system (Haberman, Brady, & Alvarez, 2015), from basic features such as size (Chong & Treisman, 2003, 2005a), color (Maule, Witzel, & Franklin, 2014), speed (Watamaniuk & Duchon, 1992), orientation (Parkes, Lund, Angelucci, Solomon, & Morgan, 2001), and number (Halberda, Sires, & Feigenson, 2006), to higher-level features such as emotion (Haberman & Whitney, 2007, 2009), identity (Fockert & Wolfenstein, 2009; Neumann, Schweinberger, & Burton, 2013), biological motion (Sweeny, Haroz, & Whitney, 2012), eye gaze (Sweeny et al., 2012; Sweeny & Whitney, 2014), and animacy (Leib, Kosovicheva, & Whitney, 2016). It is robust to variations in time (Albrecht & Scholl, 2010; Haberman, Harp, & Whitney, 2009; Hubert-Wallander & Boynton, 2015), spatial position (Alvarez & Oliva, 2009; Chong & Treisman, 2005b), and impoverished visual information (Haberman & Ulrich, 2019), and conscious access may not even be necessary (Alvarez & Oliva, 2008; Fischer & Whitney, 2011; Haberman & Whitney, 2011). Summary representation is not restricted to the central moment, as observers represent other summary statistical information, such as variance and range (Haberman, Lee, & Whitney, 2015; Lau & Brady, 2018; Solomon, 2010). Ensembles are so efficiently accessed, they tend to be the default representation when faced with memory or perceptual uncertainty about line length (Duffy, Huttenlocher, Hedges, & Crawford, 2010), time judgments (Jazayeri & Shadlen, 2010), emotional expression (Haberman & Whitney, 2009), and internal and external perceptual noise (Olkkonen, McCarthy, & Allred, 2014).

Some researchers have argued that ensemble perception, given its visual pervasiveness, may provide a source of stability in a dynamic and uncertain visual environment (Cohen, Dennett, & Kanwisher, 2016; Corbett & Melcher, 2014). While change blindness reveals a striking disconnect between what we think we see and what we can actually report seeing (Simons & Ambinder, 2005), many examples of change blindness tend to disrupt the local scene statistics while keeping global scene statistics, as outlined Oliva and Torralba (2006), intact. Recent work, however, reveals that when global scene statistics are disrupted, we are explicitly sensitive to those changes (Alvarez & Oliva, 2009), in line with the notion that we may depend on global ensemble information (akin to texture segregation) to rapidly categorize a scene (Brady, Shafer-Skelton, & Alvarez, 2017). Ensemble perception may help to maintain perceptual stability exactly because it is robust to local scene perturbations, similar to how below-threshold internal noise goes unnoticed despite its continued presence (Morgan, Chubb, & Solomon, 2008).

Although numerous studies have shown that observers possess limited knowledge regarding the individual elements of a set despite precise knowledge of the average (Chong & Treisman, 2003b; Haberman & Whitney, 2007), the average logically must be derived from information about the individuals (Haberman, Brady, et al., 2015; Neumann, Ng, Rhodes, & Palermo, 2017). However, given the instability of the individual item representations (Alvarez, 2011; Haberman & Whitney, 2012), it follows that they may be more susceptible to perceptual alteration by the more robust summary representation, even when the summary is task-irrelevant. Indeed, there is already some evidence that average size and average orientation information is hierarchically encoded such that individual judgments may impact judgments about the ensemble and vice versa (Brady & Alvarez, 2011; Utochkin & Brady, 2020). Relatedly, recent work has revealed that an ensemble of faces can influence the memory representation of an individual face within the set, such that a neutral face may be recalled as happier when presented with several other happy faces (Corbin & Crawford, 2018; Griffiths, Rhodes, Jeffery, Palermo, & Neumann, 2018).

Previous research has suggested that the interaction between the individual and ensemble might reflect an ‘optimal integration’ of information in order to minimize noise at the individual item level (Brady & Alvarez, 2011). That is, when the visual system is inundated with too much information, an efficiently derived average may be used as a guide for creating a more precise representation of a single individual. While ensembles may provide this benefit when visual working memory capacity has been exceeded, the current experiments reveal a strong influence of the ensemble on the individual even when such systems are not overly taxed. Indeed, the existence of an ensemble representation is not predicated on overwhelming working memory systems (although they may be most valuable under such conditions) — whether set information is derived automatically or in parallel is not diagnostic of ensemble representations (Whitney & Yamanashi Leib, 2018). Most critical for our purposes is whether more than one element has been integrated into a moment of central tendency.

The mounting evidence that reveals a strong influence of the ensemble on the individual should come as little surprise to individuals familiar with research on emotion perception, as it is well known that context matters when evaluating facial expressions (Barrett, Mesquita, & Gendron, 2011; Walker & Vul, 2014). Faces are rarely seen in pure isolation, and work on the ensemble perception of faces begins to address this concern by simultaneously presenting multiple faces across emotions, identities, and races. However, even these studies fall short of displaying a complete context by removing features such as hair or by presenting images extracted from a much richer scene. Indeed, recent work has demonstrated the perception of information provided by scene context with facial information obscured closely matches the perception of that scene when facial information is fully visible (Chen & Whitney, 2019). Thus, far from interfering with emotion recognition, scene context may serve to enhance it. In the current experiments, we use a stimulus set that preserves contextual information, thus we refer to multiple exemplars presented simultaneously as an ensemble of scenes. While all the images contain a visible face in some format, the additional context serves to enhance, and even hone, the perception of emotion in each stimulus.

Observers were asked to provide a valence rating for each of the scenes presented individually. Ensembles of four scenes were then constructed based on observer responses (all positively valenced or negatively valenced). Observers were asked to then provide a second valence rating of a single, cued scene from the ensemble. The only difference between the first and second viewings was the cued image’s coincidence with the three other images. To preview our results, the rating of the cued scene was pulled in the direction of the *average valence* of the irrelevant images over 70% of the time. Control experiments showed that the pull was unequivocally toward the *average* valence and not the valence of just one of the unattended items. Overall, our findings show that an irrelevant ensemble can influence the perceived valence of an individual item. This operates over high-level scene stimuli, does not result from taxing visual working memory capacity, and occurs in response to the whole ensemble, not just a single, subsampled item.

## Experiment 1

### Method

#### Participants

Fifty Rhodes College undergraduates, ages 18 to 22, participated in this study for course credit. All participants gave informed consent and had normal or corrected-to-normal vision. This research, and all research described herein, was conducted in accordance with the Declaration of Helsinki and was approved by the Institutional Review Board at Rhodes College.

#### Stimuli and Design

Experiments were implemented using custom scripts written in Psychtoolbox (Brainard, 1997) within Matlab. Each experiment was divided into two parts, completed in a single experimental session. Stimuli were images depicting complex scenes of varying emotional valence, collected from advertisements appearing in the *American Journal of Psychiatry* during the 1950s for a separate research project. Stimuli were displayed on a Dell flat screen monitor at a resolution of 1920 × 1080. In Part I, all images were 600 × 600 pixels subtending 16.2° × 16.2° of visual angle, displayed in the center of the screen. In Part II, an ensemble of four images, each 280 × 280 pixels subtending 7.6° × 7.6° of visual angle, appeared in a square formation with each image displayed 3.53° degrees radially from the center. These sets were constructed from the same images observers rated in Part I.

#### Procedure

**Fig. 1:**
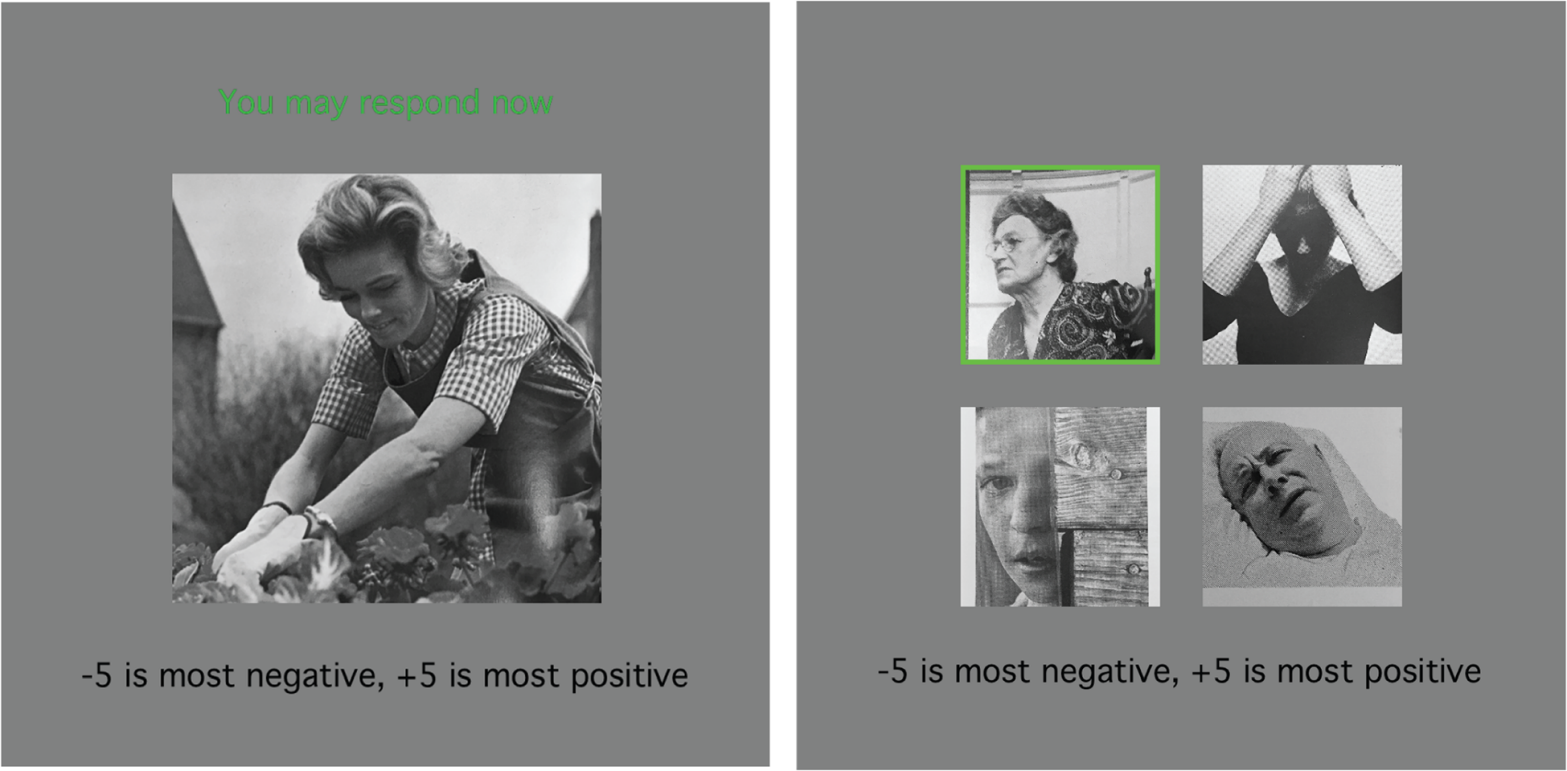
Example trials from Part I (*left*) and II (*right*). Participants rated the emotional valence of a single image in Part I and then a cued image from a set of four in Part II

Participants sat 63 cm from the screen. In Part I, they viewed a total of 190 unique images presented in random order. Each image was shown for at least 1.5 seconds, at which point they were instructed to rate the image’s emotional valence on a scale from − 5 (negative) to + 5 (positive), excluding 0 (Fig. 1). In Part II, participants viewed the same images in sets of four, with each image in the set being unique. After 1 second, participants were cued to one of the four images at random by highlighting the target image with a green border. After an additional 500 ms, the images disappeared and the participants were instructed to rate the emotional valence of the cued image, just as they did in Part I. Sets consisted of either only positive images or negative images based on the ratings given in Part I. Each participant performed 200 trials in Part II.

We chose to fix the order of the blocks (Part I: rate individual images followed by Part II: rate those images again when surrounded by an ensemble) because it preserved critical individual differences. Part II is tailored to each observer’s perceptual experience. While we acknowledge potential concerns associated with a fixed serial order, the alternative, where an independent sample for each part of the experiment is used, creates even more problems. For example, averaging the ratings of individual images results in regression to the mean, whereby the functional range of the valence ratings is drastically reduced. Since Part II depends on the ratings of Part I, this restricted range would make it impossible to generate ensembles that were sufficiently different from the target image. Thus, we opted for the fixed order in order to preserve individual differences across valence ratings.

### Results and Discussion

**Fig. 2:**
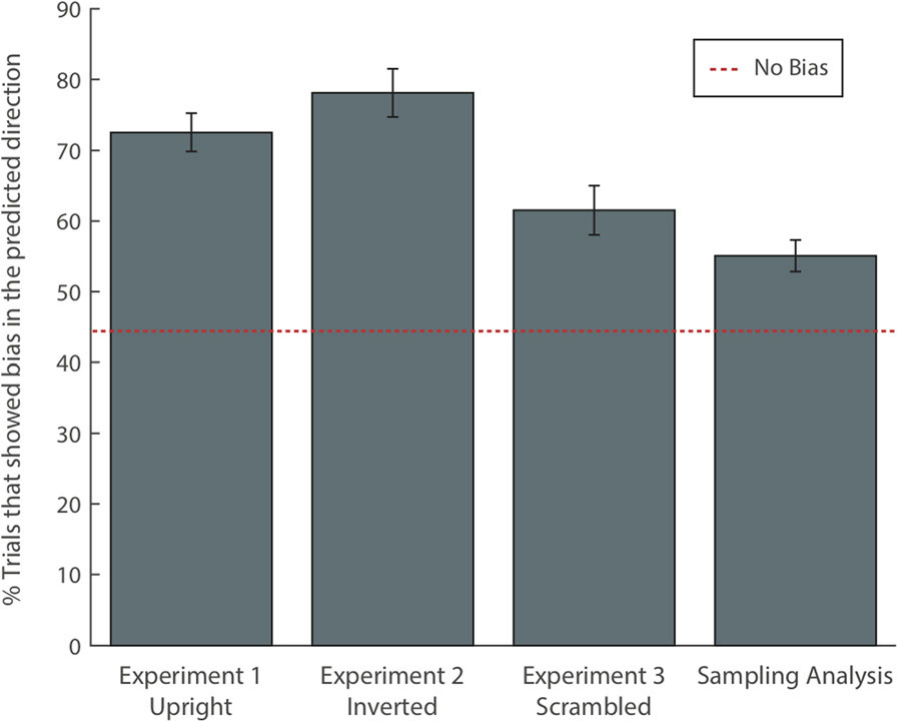
Percent of trials in which emotional bias was in the expected direction. Bias in the expected direction was calculated by determining the percent of cued image ratings that shifted toward the mean of the distractor image ratings. ‘No Bias’ line determined by Monte Carlo simulations. Error bars represent one standard error of the mean

For each participant, we calculated the proportion of trials in which the emotional rating of the cued image in Part II shifted in the direction of the ensemble (i.e., the average valence of the other three items given by the participant in Part I). We only included trials in which the difference between the mean emotion of the irrelevant ensemble and the original rating was greater than 0.5 in order to ensure there was a perceptually meaningful difference that could create a shift. On average, the rating of the cued image was shifted toward the irrelevant ensemble on 71.4% of trials (M_Bias_ = 0.71, SD_Bias_ = 0.06, t(49) = 8.12, *p* < 0.001), compared to 65% chance performance as determined by Monte Carlo simulations, which simulated 10,000 observers selecting target responses at random for Part II; Fig. 2.

Note that the simulations predicted 65% of the trials would be pulled in the direction of the irrelevant ensemble just by chance. The reason this number is so high is due to the statistical skew of the distribution. For example, if an observer rated an image in Part I as + 5 in valence, it will always be true that the second rating in Part II will be the same or less than + 5, since responses cannot be > 5. Therefore, it is difficult to determine the pull of the irrelevant ensemble independent of the response bias introduced by our experimental design. To address this, we ran a secondary, more conservative analysis designed to remove the response bias. We included only trials in which the original rating of the image from Part I was *exactly* ± 3, which is the midpoint of the response distribution. This approach allowed us to isolate the influence of the irrelevant ensemble. This control analysis resulted in an average of 20 trials per participant included in the analysis (although this resulted in the inclusion of only 10% of the data per observer, the large sample size overcame any power concerns, as revealed by the statistical results below). On average, participants’ ratings of the cued image shifted in the direction of the mean ensemble rating on 73% of these trials, comparable to what was found in the original analysis (M_Bias_ = 0.73, SD_Bias_ = 0.19, t(49) = 8.42, *p* = < 0.001, compared to 45% chance performance, as determined by Monte Carlo simulations). The impact of including only the ratings of ±3 was primarily on the Monte Carlo simulations, which now showed chance responding (i.e., no bias) at 45%. With the potential response bias removed, we have created a purer measure of the influence of the irrelevant ensemble. We used this approach for all subsequent experiments. If emotional information of the cued images in Part II was processed independently of the other items in the set, we would expect no consistent bias of the ratings of images in Part II relative to Part I. However, the emotional rating of the cued image was pulled toward the mean emotional rating of the surrounding images on a significant proportion of the trials. These results suggest that high-level, irrelevant emotional information consistently influences the perceived emotion of individual scenes.

This bias is not simply the result of the influence of a single, random item from the irrelevant ensemble, but rather reflects its aggregate influence. We once again conducted the bias analysis described above, but instead of determining the influence of the entire ensemble on the rating of the cued image, we determined the influence of a single, randomly selected item from the set. As before, these calculations were based on participants’ ratings from Part I of the experiment. For each observer, we averaged the results of 5000 iterations of the sampling procedure to obtain the average pull of a single image from the set. Only 56.6% of the sampled trials were pulled in the direction of the irrelevant ensemble, averaged across observers. This was significantly above chance (t(49) = 4.6, *p* < 0.001) suggesting a single irrelevant item affected the target valence rating. Critically, however, the random sample exerted significantly less pull on that rating than when the whole ensemble was considered (t(49) = 4.43, *p* < 0.001). This analysis demonstrates that the entire ensemble, not just a randomly sampled image within the ensemble, is responsible for influencing the perceived emotion of the cued image.

We then calculated the magnitude of the pull on those trials that demonstrated a pull toward the mean. On average, the difference between the irrelevant ensemble rating (rated in Part I) and the original rating of the cued image (also rated in Part I) was 0.61, and the difference between the original rating of the cued image (rated in Part I) and the new rating of the cued image (rated in Part II) was 0.1. This means that the shift of the ratings was around 16% of the maximum possible, an impressive shift given observers were not supposed to be responding to the uncued stimuli.

## Experiment 2

Experiment 1 established that perceived emotion of an individual scene can be influenced by an irrelevant surrounding ensemble. Additional analyses demonstrated that randomly sampled, individual images did not match the bias effect revealed by the average of the entire ensemble. Experiment 2 replicated Experiment 1 using inverted ensemble images while keeping the target image upright. Inverting images decreases recognition of faces while keeping individual part information intact (Farah, Wilson, Drain, & Tanaka, 1998; McKone, Martini, & Nakayama, 2001), supporting the notion that faces are processed configurally. Expression recognition is also impaired for both single faces (Bartlett & Searcy, 1993) and ensembles of faces (Haberman & Whitney, 2007). We therefore hypothesized that the emotional pull of the inverted ensemble images would be weaker than in Experiment 1.

### Method

#### Participants

Nineteen Rhodes College undergraduates, ages 18 to 22, participated in this study for course credit. All participants gave informed consent and had normal or corrected-to-normal vision.

#### Stimuli and design

**Fig. 3:**
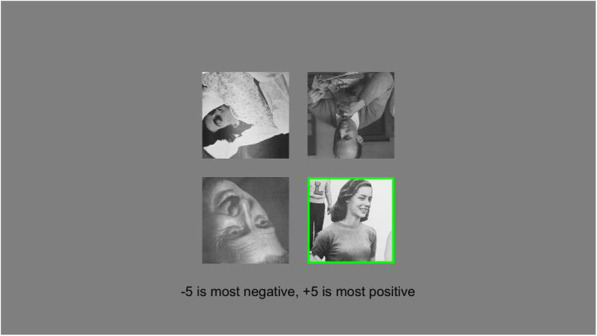
Example trial from Part II. Participants saw an upright image that was cued after 1 second along with inverted distractor images

The setup for Experiment 2 was identical to that of Experiment 1, except that ensemble images in Part II were inverted, while the target image remained upright (Fig. 3).

#### Procedure

The experimental procedure was identical to that of Experiment 1.

### Results and discussion

The bias analysis was conducted as described in Experiment 1. Similar to Experiment 1, participants’ ratings of the cued image shifted in the direction of the mean emotional rating of the ensemble (M = 0.78, SD = 0.14, t(18) = 69.71, *p* = < 0.001, compared to 45% chance performance determined by Monte Carlo simulations). Emotional pull persisted despite the inversion of the ensemble images, suggesting that ensemble valence information from the inverted scenes still influenced the perceived valence of the cued scene. Additionally, the amount of bias for the inverted condition likely came from the same population distribution as the upright condition, as indicated by a Bayesian independent samples *t* test implemented in JASP (BF_01_ = 2.14; Jarosz & Wiley, 2014)). Although these results seem to contrast our initial hypothesis, which was based on the ensemble literature (Haberman & Whitney, 2007), accurate emotional processing is still possible when relying just on component information, which is still available with inverted stimuli (Lipp, Price, & Tellegen, 2009; McKelvie, 1995). In previous ensemble work, observers were explicitly asked to extract the average expression of a set of inverted faces, a task that relies upon precise representations. In the current task, it is possible that access to the overall valence of a set of scenes, which may not require the same level of precision required in previous ensemble tasks, remains robust in the presence of inversion. The bias effect is as large as it was in Experiment 1, a testament to the power of averaging, even when accomplished unintentionally.

## Experiment 3

Experiments 1 and 2 demonstrate that an ensemble can bias the perceived emotion of an individual image, regardless of the ensemble orientation. Experiment 3 further pushes the limits of unintentional averaging, testing whether the bias effect is mitigated when semantic content is disrupted by scrambling the ensemble images. We hypothesized that there would be little to no emotional pull of the cued image toward the average emotional valence of the ensemble.

### Method

#### Participants

Thirty-one Rhodes College undergraduates, ages 18 to 22, participated in this study for course credit. All participants gave informed consent and had normal or corrected-to-normal vision.

#### Stimuli and design

**Fig. 4:**
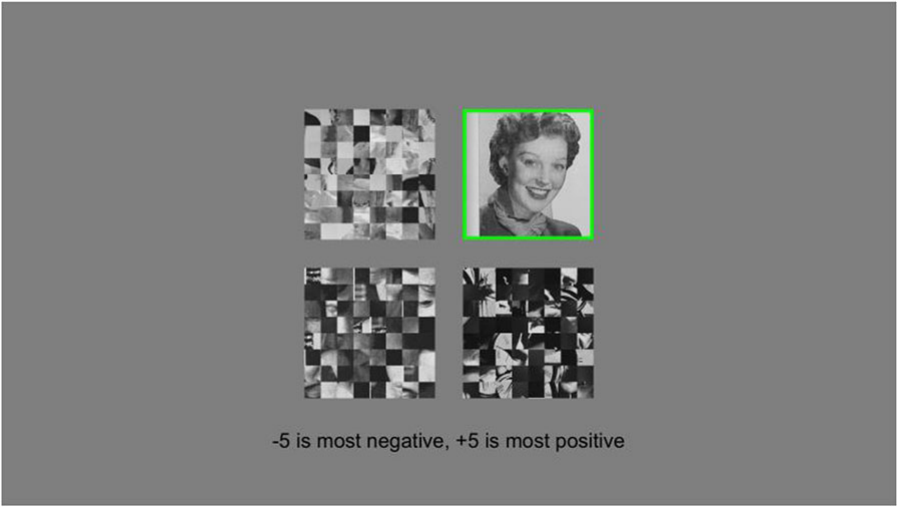
Example trial from Part II. Participants saw four scrambled images, one of which was unscrambled and cued after 1 second

The setup for Experiment 3 was identical to those of Experiments 1 and 2 except that all images in Part II were scrambled prior to running any participants, ostensibly removing high-level, semantic content from the images while preserving low-level image features. The scrambled images used were the same across participants. On each trial, participants viewed four scrambled images, and after 1 second, the target image was unscrambled and cued with a green border while the other images remained scrambled (Fig. 4). The set remained on the screen for 1.5 seconds before disappearing, at which point the participants were instructed to rate the emotional valence of the cued image.

Stimuli were scrambled by dividing the images into 8 × 8 grids and randomly permuting the arrangement of the tiles.

#### Procedure

The experimental procedure was identical to those of Experiments 1 and 2 except for the use of scrambled images.

### Results and Discussion

A bias analysis was conducted as described in Experiment 1. Participants’ ratings of the cued image was shifted toward the mean emotion of the surrounding scrambled images (M = 0.62, SD = 0.19, t(30) = 4.74, *p* < 0.001, compared to 45% chance performance as determined by Monte Carlo simulations; Fig. 2). However, the effect of the scrambled ensemble was significantly weaker compared to the upright images shown in Experiment 1, as determined by an independent samples *t* test (t(79) = 2.52, *p* = 0.014).

**Fig. 5:**
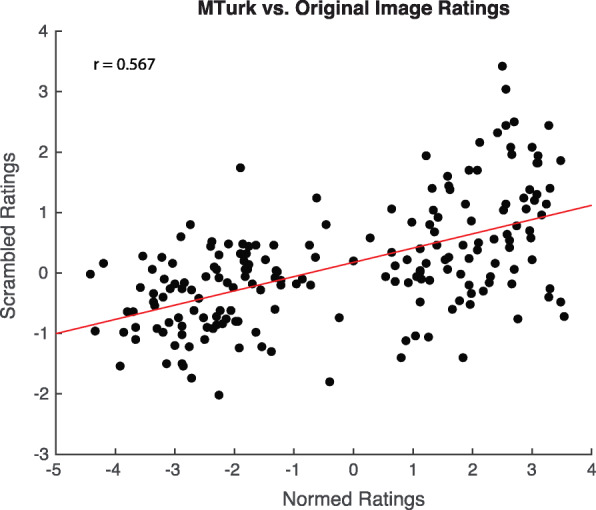
Correlation between the average ratings of the original images (*x-axis*) and the average ratings of the scrambled images (*y-axis*)

We were somewhat surprised to find a significant pull toward the ensemble of the scrambled images, given the disruption of the original images’ configuration. We explored the possibility that some high-level emotional content was still available in the scrambled images in a study run on Mechanical Turk. We created 19 sets of 10 pseudorandomly selected scrambled images from our original set of 190. Fifty participants (who were consented and compensated for their time) each rated one set of 10 scrambled images.^1^ We then correlated these ratings with the average ratings of the original images from Experiment 1. This analysis revealed a relatively strong correlation between the ratings (*r* = 0.56; *p* < 0.001; Fig. 5) suggesting that some valence information was available even when the images were scrambled. It is important to note, however, the significant reduction in the overall pull is likely due to the removal of the much of the original configural content of the image. Overall, these results confirm that emotional valence of the ensemble images, not just low-level features (which were preserved in the scrambled images), are responsible for biasing the perceived emotion of the cued image.

It is conceivable that, since the scrambled images contain reduced discernable high-level scene information, the memory for the original rating of a given image might be easier to access. Note, however, that this account would still be consistent with our hypothesis that the ensemble is driving the bias observed in Experiment 1, and that no discernable ensemble is available when viewing scrambled images in Experiment 3. To test the possibility that the memory for target images is better in the scrambled version (Experiment 3) than in the intact version (Experiment 1), we compared the absolute magnitude of the difference between the target ratings in Part II and Part I in each version (intact and scrambled). Specifically, if memory for the target were better in Experiment 3, we would expect the rating of the second image to be more consistent with the rating of the first image across all trials (i.e., less of a difference). We did not observe this in our analysis. The overall absolute difference between the first and second ratings of target images was nearly identical across Experiments 1 and 3 (*p* > 0.05), suggesting that in Experiment 3 observers’ responses were distributed both toward and away from the ensemble (i.e., unaffected by it), and not that memory for the target was better because of reduced interference from the surrounding images.

### General discussion

These experiments convincingly reveal an unintentional influence of an irrelevant ensemble on the perception of an individual item. When observers were asked to evaluate the valence of an individual scene, their ratings shifted toward the average of the other scenes in the set. The magnitude of this shift was around 16% of maximum, maximum being defined as the unlikely event of altogether ignoring the cued image and instead reporting only the average of the three irrelevant items. This shift occurred in spite of potential anchoring effects (Helson, 1964), as observers had already evaluated each scene once, originally presented in isolation. The pull of the average persisted even for inverted irrelevant scenes, suggesting average valence information was still available and could wield an influence on the individual. When high-level valence information was disrupted by scrambling the irrelevant scenes, observers’ assessments of the cued (intact) scene was only marginally influenced, suggesting the information driving the unintended shift is high-level in nature.

While it is the case that observers potentially had time to saccade to all images in a given set, it is nonetheless surprising that attention failed to fully suppress the irrelevant information. Once the target was highlighted, attention might be expected to suppress information in other spatial locations (Smith, Singh, & Greenlee, 2000), but some information clearly leaked through. What is most compelling, however, is the form of the information that bypassed the attentional gate. It was not just that a single item influenced the second rating of the target image, but rather it was the whole *ensemble*, as revealed by our sampling analysis. Thus, attention might fail to suppress irrelevant ensemble information, even when observers are not told anything explicit about an ensemble code.

It is interesting to note that individual ratings were pulled in the direction of the ensemble even within an emotional category. All sets viewed in Part II contained entirely positive or negative images, based on the observer’s own ratings, which makes the strong connection between the individual item and the irrelevant ensemble even more compelling. It did not require a strongly negative irrelevant set to affect a positive item. Positive sets whose irrelevant ensemble had a valence greater than three pulled the second ranking of the individual in the same direction, and positive sets whose irrelevant ensemble had a valence less than three did as well. The same was observed for the negatively valenced irrelevant ensembles.

Ensemble perception has been demonstrated for a number of high-level stimuli, including biological motion (Sweeny et al., 2012), animacy (Leib et al., 2016), and gaze (Sweeny & Whitney, 2014), but this is one of the first examples demonstrating average scene perception. The fact that it operated without explicit instruction to engage in any sort of averaging process is a testament to the power of ensemble perception. That said, the judgments observers made about these scenes concerned their valence, which arguably is another version of an average expression judgment, and not the sort of characteristic typically evaluated when viewing scenes (e.g., openness; Greene & Oliva, 2009). Interestingly, a recent poster presentation revealed precise ensemble perception for scenes when observers were asked to judge scene content (naturalness) and boundary (openness) information (Tharmaratnam, Haberman, & Cant, 2019, which may be more directly linked to traditional measures of scene perception (Greene & Oliva, 2009; Oliva & Torralba, 2001).

These results are striking because the shift toward the irrelevant ensemble occurred for ratings on stimuli observers already had rated when viewed in isolation. In other words, the shift indicates that the influence of the irrelevant ensemble was so powerful that it overcame traditional anchoring effects (Helson, 1964). One might reasonably expect the first and second ratings of the same image to be unchanged, especially given that observers were likely to remember having seen each image (Konkle, Brady, Alvarez, & Oliva, 2010). In spite of likely having remembered seeing a given image and giving it an initial rating, participants’ second ratings of the cued image were strongly influenced by the *ensemble* rating of the other images.

Often times ensemble perception is thought to operate optimally given overwhelming working memory conditions. When the visual system is inundated with information, or when it is unable to discriminate individual items, the average may be a reasonable proxy for any given single item (Brady & Alvarez, 2011; Parkes et al., 2001). In the current experiments, however, there are no working memory pressures and all items are easily distinguishable, and yet ensemble information wields a strong influence on individual item representation. Contextual information can certainly influence judgments about facial expression (Barrett et al., 2011), but our results reveal that the influence is coming from an inadvertently derived ensemble, and not just a single, randomly sampled item from the periphery.

Overall, these findings converge with and extend the work of Griffiths and colleagues (Griffiths et al., 2018), who showed that a crowd of faces can alter the memory representation of an individual face. Our results confirm that the influence is high-level in nature, and relates to the ensemble representation itself. They critically show that the ensemble can influence the individual item representation even when working memory systems are not operating at capacity (i.e., observers only had to ever remember one item).

## Conclusion

Ensembles are an efficient way to compress a remarkable amount of information about the visual world. They enhance cognition by granting access to more information than might be predicted by the fidelity of individual object representation (Alvarez, 2011), and they facilitate rapid, global scene perception (Brady et al., 2017). While some work suggests that ensembles implicitly operate when working memory demands are high (Brady & Alvarez, 2011; Griffiths et al., 2018), providing stability in a noisy and dynamic environment, our results reveal that high-level ensemble scene information can influence individual item representation even when memory demands are low. The influence is substantial — more than 71% of the trials were pulled in the direction of the irrelevant scenes — and is unequivocally the result of the *ensemble*, not just a randomly selected item from the set. Overall, our results suggest that the surrounding context can alter a perceptual representation in meaningful ways, even when counter to current task goals. Practically, unintentional averaging could lead to inaccurate interpretation of emotional information, so perhaps it is best practice to hold serious, one-on-one conversations in more secluded settings.

## Data Availability

All data are available on the Open Science Framework at the following link: DOI 10.17605/OSF.IO/S35NJ

## Footnotes

1 Participants were not prevented from rating more than one set of the scrambled items.
